# Diagnostic and Therapeutic Pitfalls Encountered in a Young Adult Patient with a Symptomatic Chronic Subdural Hematoma Mimicking a Subacute Epidural Hematoma in the Presence of a Galassi Grade III Arachnoid Cyst: Case Report

**DOI:** 10.3390/reports9020174

**Published:** 2026-06-04

**Authors:** Marios Theologou, Nikolaos Syrmos, Vaitsa Giannouli

**Affiliations:** 1 Department of Neurosurgery, General Hospital of Thessaloniki Georgios Papanikolaou, 57013 Thessaloniki, Greece; milanako76@yahoo.gr; 2 School of Medicine, Aristotle University of Thessaloniki, 54124 Thessaloniki, Greece; 3 Department of Psychology, Democritus University of Thrace, 68300 Didymoteicho, Greece

**Keywords:** intracranial, hematoma, cognitive assessment, surgery, minimally invasive

## Abstract

**Background and Clinical Significance**: Chronic subdural hematomas (cSDHs) present characteristic imaging findings, making the diagnosis straightforward. In rare cases, arachnoid cysts (ACs) may be associated with their formation. There is still no consensus regarding their treatment; **Case Presentation:** A young adult male presented with occipital headache. Neurological examination was normal. Laboratory investigations were within physiological limits. A CT scan revealed the presence of a Galassi Grade III temporo-parietal AC accompanied by a parietal epidural hematoma (EDH) on the right side. His medical history was significant for treated hypertension. There was no use of anticoagulants, antiplatelets, or history of trauma. Vascular pathology was excluded by MRA/MRV. He was discharged for home care and was readmitted 10 days later after a repeat CT scan. A brief cognitive assessment with the Mini-Mental State Examination (MMSE) revealed mild cognitive impairment. A burr-hole evacuation was performed, and a drainage catheter was left in place for 24 h. Intraoperative findings were consistent with a chronic subdural hematoma. The patient was discharged with complete resolution of symptoms. A follow-up CT scan performed one month postoperatively confirmed the favorable result. Cognitive functions were normal on follow-up; **Conclusions:** A SDH may mimic the characteristics of an EDH in the presence of an AC. The most common symptom is cephalalgia. Neurocognitive impairment may occur secondary to elevated intracranial pressure. A burr-hole hematoma evacuation may be sufficient. Further treatment should be considered only in the case of complications associated with ACs.

## 1. Introduction and Clinical Significance

cSDHs are commonly detected in elderly patients after recent (>14 days) head trauma. Imaging findings are typically characteristic, making the diagnosis straightforward [1]. In rare cases, ACs may be associated with the formation of cSDHs [2]. The demographic characteristics of these patients differ significantly compared to the previous population, as they are young adults with no history of head trauma. These cases are rare, and the published results are scarce. Thus, there is still no consensus regarding the optimal treatment strategy. We present a case of a symptomatic cSDH mimicking a subacute epidural hematoma (sa-EDH) in a patient with a Galassi grade III AC, discussing the diagnostic and treatment challenges alongside the demographic and clinical characteristics of these patients.

## 2. Case Presentation

A 47-year-old Caucasian male presented to the emergency department with a moderate-intensity occipital headache (lasting > 72 h), which aggravated during routine physical activity and showed no improvement with usual treatments. He reported no nausea, vomiting, photophobia or phonophobia. Neurological examination was normal. Laboratory investigations were within physiological limits, including coagulation tests. A CT scan was performed, revealing the presence of a previously known Galassi Grade III right temporo-parietal arachnoid cyst with a suspected subacute (isointense) parietal EDH in close association with the cyst’s wall (Figure 1A,B, Figure 2A and Figure 3). The patient had a history of treated hypertension (Amlodipine and Valsartan), with no use of anticoagulants or antiplatelets, while the possibility of trauma was excluded for a minimum period of 30 days. He also mentioned regular vaping (60 mL/week, nicotine mixture 6 mg/mL) and occasional smoking. The patient was admitted, and an MRI (Figure 2A and Figure 4A) with MR angiography and MR venography was performed, which excluded vascular pathology. After being followed up for 48 h, he was discharged for home care, with a planned readmission 10 days later. A repeat CT scan was performed. The volume estimation, employing the Region of Interest (ROI) Volume Computing Tool of the Horos Project v3.3.6 DICOM Viewer, revealed that the collection had a total volume of 30.82 cm^3^. On re-evaluation, he complained of forgetfulness. Thus, a Mini-Mental State Examination (MMSE) was conducted, resulting in an overall score of 23, pointing toward mild cognitive impairment. A burr-hole was placed under local anesthesia, using measurements acquired from the zygoma, the acoustic meatus, the inion and the mid-line, targeting the center of the finding. Visualization of the dura, without evidence of an epidural collection, immediately called the initial diagnosis of epidural hematoma into question. An incision with a No. 11 blade revealed a subdural hematoma membrane, which was opened using bipolar cautery, and a reddish-brown fluid was released under pressure. The burr-hole was irrigated with approximately 200 mL of Physiological Saline Solution before placing a catheter inside. A repeat CT scan was performed 24 h post-surgery, and the drainage catheter was removed. The early post-treatment period was uneventful. He was discharged the next day with complete resolution of the pre-surgery symptoms and without any new findings during the neurological evaluation. A one-month postoperative follow-up CT scan confirmed the favorable result of the treatment (Figure 4B). Cognitive functions improved according to the MMSE score, which was 29 at the one-month follow-up. The patient returned to his everyday routine 2 weeks post-surgery. He was clinically assessed in a standard medical follow-up schedule (months 1, 3, 6, 12) and was symptom-free during that time.

## 3. Discussion

Epidural hematomas (EDHs) are described as biconvex or lens-shaped lesions on neuroimaging, causing a mass effect and thus an increase in intracranial pressure [1]. The shape is attributed to the attachment between the dura and the inner table of the bone, which is stronger in those >60 years old, significantly lowering the incidence of presentation. The temporal region is affected in approximately 75% of cases, mostly after significant head trauma, causing either the mechanical detachment of the dura from the bone or a vascular injury, resulting in continuous blood flow that dissects the anatomical structures. Other localizations are uncommon, and if detected, a screening for vascular malformations should be performed, similar to the presented case. According to the imaging characteristics and the demographic group of the patient, the lesion was attributed to an EDH. It has been previously reported that an organized/encapsulated SDH may mimic the imaging characteristics of an EDH [3,4]. However, the presence of lobulations and/or calcifications would be expected in this case. Elevated intracranial pressure may restrict the space available for hematoma expansion. Therefore, the presence of an AC or other space-occupying lesions may alter hematoma morphology and complicate radiological diagnosis. The isodense presentation is characteristic of the process of gradual degradation of an older hematoma, and in the absence of any vascular malformation and notable trauma, the collection was attributed to a spontaneous subacute SDH formed 3–14 days prior to the evaluation. In the absence of any clinically significant symptoms apart from headache, the patient was discharged for home care with a plan for reevaluation and potential evacuation after 10 days, once the hematoma had evolved into a more liquefied chronic collection [5], as this could potentially provide a more favorable result, demanding a less invasive procedure (burr-hole vs craniotomy).

ACs are benign congenital malformations that account for approximately 1% of all intracranial space-occupying lesions [6], with a significant male predominance [7]. They are classified according to the Galassi system [8] and may present a notable mass effect, causing significant midline shift, similar to this case. They are rarely symptomatic; thus, they are mostly diagnosed as an incidental finding on neuroimaging [9]. The presence of an AC is associated with an increased incidence of subdural hematomas (SDHs) in children, juveniles and young adults [2,10,11,12,13,14,15]. The pathophysiological mechanisms resulting in this statistical correlation are not yet completely understood, and no definitive association with any risk factor, including cyst size, has been defined, probably due to the restricted number of patients resulting in sporadic case reports or small case series presented in the literature. Theoretically, bridging veins passing through the arachnoid cyst, the presence of vessels on the outer layer of the AC, or even the lower compliance of the cystic formation when compared to the adjacent brain tissue may potentially predispose the formation of the hematoma, even after insignificant trauma that is often overlooked and thus not reported in the anamnesis. We should emphasize that the aforementioned statement is just a hypothesis based on the blood supply anatomy [16]. Literature highlights the association between the AC localization and the incidence of hematoma formation, as they seem to be more frequent in patients with middle fossa findings [15], which is consistent with our case.

The patient complained of an intensifying headache, while the neurological examination was normal. It is known that elevated intracranial pressure may result in the manifestation of various neurological symptoms. Interestingly, at the time of diagnosis, these cases seem to present with headache as their only symptom [14]. In addition, our patient presented mild cognitive impairment on readmission. Despite the inability to directly associate the hematoma localization with cognitive function areas, his cognitive status may have been affected by elevated intracranial pressure. Intracranial pressure homeostasis in these patients may be more susceptible to disruption. It should be highlighted that the elevated intracranial pressure described is only a logical assumption based on the authors’ assessment. A direct, invasive ICP measurement was not performed, as it was not deemed medically necessary for the patient’s management or ethically justified.

Even though some cSDHs may resolve spontaneously, the consensus is that surgical treatment should be considered in all symptomatic patients [17]. The optimal surgical strategy in these cases is controversial. Some authors advocate drainage through a burr-hole, while others believe that this should be combined with the partial or even total removal of the AC wall, demanding a more aggressive approach. Our surgical team opted for the less invasive approach consisting of hematoma evacuation alone. We believe that this approach can ensure the safety of the patient, while avoiding more invasive procedures. Also, it is our strong belief that the intracranial pressure equilibrium will not be disrupted by the procedure unless the cyst wall is penetrated, in which case we would probably perform a cysto-peritoneal shunt or conduct an endoscopic cysto-cisternal fenestration, as these minimally invasive procedures provide favorable results, while minimizing complications [18]. The craniotomy would be considered only as a last resort treatment, as the use of less invasive approaches is preferable [19]. The use of intraosseous needles has been proposed as a less invasive alternative for hematoma decompression [20]. However, we do not advocate techniques that omit irrigation, as current evidence suggests improved outcomes when irrigation is performed [21]. Other minimally invasive strategies, including middle meningeal artery embolization, have also been reported, particularly in recurrent cases [22,23]. We should highlight that the overall treatment strategy in these patients is challenging, similar to any case requiring management of CSF pressure homeostasis, which can be associated with the lack of understanding of the homeostatic mechanisms [24].

## 4. Conclusions

A SDH may manifest imaging characteristics of an EDH in the presence of an AC. These patients may present with various symptoms, headache being the most common. Neurocognitive impairment may also occur secondary to elevated intracranial pressure. A burr-hole hematoma evacuation without surgical treatment of the arachnoid cyst may be sufficient. Further treatment should be considered only in the case of complications associated with the AC.

## Figures and Tables

**Figure 1:**
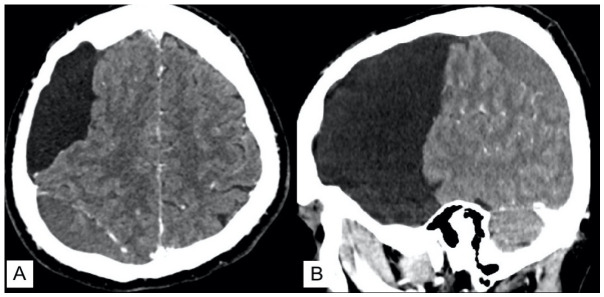
Neuroimaging: (**A**) axial and (**B**) sagittal pre-surgery CT with contrast, depicting a part of the Galassi III AC on the right side and a collection initially attributed to an EDH.

**Figure 2:**
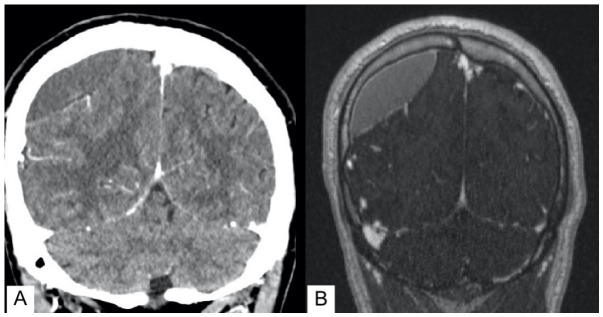
Neuroimaging: (**A**) coronal pre-surgery CT with contrast and (**B**) MRI-MRA/MRV depicting the lentiform lesion.

**Figure 3:**
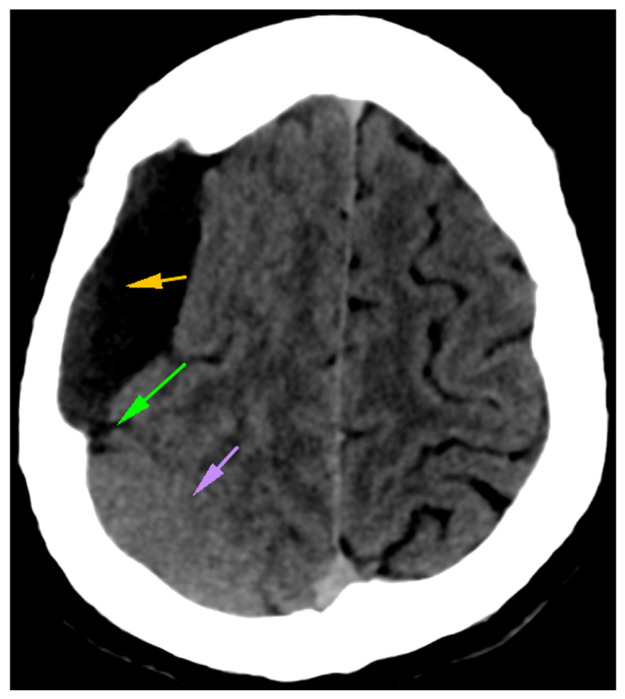
Orange arrow—arachnoid cyst. Green arrow—touch point between the cyst and lentiform lesion. Purple arrow—isodense lentiform lesion of the right parietal region.

**Figure 4:**
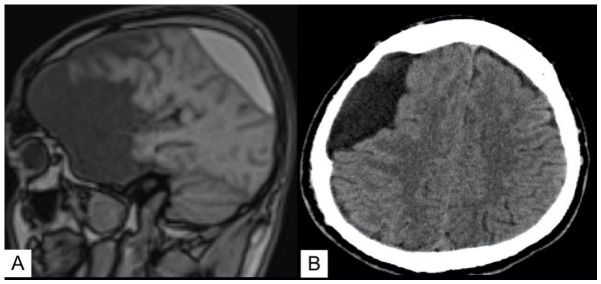
(**A**) Sagittal MRI of the suspected EDH. (**B**) post-surgery CT scan showing a complete resolution of the hematoma at the final follow-up.

## Data Availability

The original contributions presented in this study are included in the article. Further inquiries can be directed at the corresponding authors.

## Abbreviations

The following abbreviations are used in this manuscript:

EDH	Epidural Hematoma
SDH	Subdural Hematoma
AC	Arachnoid Cyst
MMSE	Mini-Mental State Examination
CT	Computed Tomography
MRI	Magnetic Resonance Imaging
MRA	Magnetic Resonance Angiography
MRV	Magnetic Resonance Venography
